# The effect of outpatient antibiotic treatment of coronavirus disease 2019 on the outcomes in the emergency department: a propensity score matching study

**DOI:** 10.3325/cmj.2022.63.53

**Published:** 2022-02

**Authors:** Armin Atić, Maša Sorić, Josip Stojić, Ana Andrilović, Vlatko Grabovac

**Affiliations:** Emergency Department, Dubrava University Hospital, Zagreb, Croatia

## Abstract

**Aim:**

To determine the effect of outpatient oral antibiotics on coronavirus disease 2019 (COVID-19) in patients presenting to the emergency department.

**Methods:**

This retrospective cohort study with propensity score matching conducted at University Hospital Dubrava collected data on all emergency department visits due to COVID-19 in November 2020. The primary outcome was hospital admission. The secondary outcomes were pneumonia development, respiratory failure, and required level of respiratory support.

**Results:**

Overall, 1217 visits were evaluated and 525 patients were included in the analysis. After propensity score matching, 126 pairs of treated patients and controls were identified. Patients and controls did not differ in physical examination findings, laboratory test results, radiographic findings, or defined outcomes before and after matching.

**Conclusion:**

This study suggests no benefit of empirical oral antibiotics for outpatient treatment of COVID-19. In patients presenting to the emergency department, prior oral antibiotic treatment did not affect hospital admission rates or the level of respiratory support required.

The coronavirus disease 2019 (COVID-19) pandemic has increased antibiotic prescription rates in the community, which could negatively affect antimicrobial stewardship and resistance (1,2). Up to 71% of COVID-19 patients had an antibiotic administered without a clinical indication (3,4). The reasons behind antibiotic prescription for a known or suspected viral disease are manifold. First, during the influenza pandemic, the rates of bacterial super-infections were higher (5). Second, the usual clinical signs of COVID-19 overlap with the signs of bacterial disease. Finally, drugs such as azithromycin were postulated to play a role in treating COVID-19 due to their antiviral and immunomodulatory activities (6,7). An expected benefit from outpatient antibiotic treatment is preventing a more severe disease and hospital admission. Reducing hospital admission rates during pandemics would alleviate the overload of the health care system, particularly during pandemic surges.

Guidelines for treatment of COVID-19 issued by the Croatian Ministry of Health and the European Medical Agency in late 2020 did not include administration of empirical antibiotics (8,9). Despite the guidelines, latest data available in Croatia show an increase in the prescription of azithromycin in clear correlation with the increase of COVID-19 patients (10). However, reported rates of bacterial co-infections in COVID-19 patients are low. A meta-analysis by Langford et al (11) demonstrated that only 5.9% of all patients hospitalized for COVID-19 had bacterial co-infections, while the majority of patients had received antibiotics at some point in their treatment. Recently published results of the PRINCIPLE Trial Collaborative group showed that azithromycin did not reduce the number of hospital admissions or deaths compared with usual care in the general population with suspicion of COVID-19 or a proven COVID-19 infection (12). The proportion of patients seeking hospital attention in the PRINCIPLE trial is low, and conclusions drawn for this population are possibly underpowered.

Another concern with antibiotic overuse are adverse effects of antibiotics. In a recent randomized controlled study (RCT) comparing azithromycin with placebo, early after starting therapy with azithromycin, the treatment group had more gastrointestinal symptoms, including diarrhea, abdominal pain, and nausea than the placebo group (13).

Previously published studies involving asymptomatic patients or specific age groups had a significant loss to follow-up and, most importantly, had a low incidence of emergency department (ED) attendance or hospital admission. In our study, we investigated the effect of prior oral antibiotics as empirical treatment in all adult COVID-19 patients who presented to the emergency department. The primary outcome of interest was hospital admission; secondary outcomes were pneumonia development, respiratory failure, and need for respiratory support.

## PATIENTS AND METHODS

We retrospectively reviewed the medical records of all emergency department visits at a COVID-19 hospital in University Hospital Dubrava that took place from November 1 to December 1, 2020. University Hospital Dubrava was temporarily appointed the chief hospital for the acute care of exclusively COVID-19 patients. All patients presented with a positive polymerase chain reaction (PCR) or rapid antigen test (RAT) for SARS-CoV-2 infection. Therefore, in the studied period no testing was performed at our ED. The study was approved by the Ethics Committee of University Hospital Dubrava (2021/2503-05). Written or verbal consent was waived.

### Data collection

Data were collected during March 2021 by three independent researchers from the hospital information system by IN2 group (iBIS, IN2, Zagreb, Croatia). Data included demographic information, clinical examination results, laboratory test results, and radiographic findings. The data were further independently validated by two senior researchers, and the differences were adjudicated by discussion. The data were obtained during routine clinical examinations and not for the purposes of the study.

### Patient eligibility

We enrolled all adult patients presenting to our ED from November 1 to November 30, 2020. Patients excluded were those with a history, physical examination, laboratory findings or imaging highly suggestive of a bacterial infection (eg, abscesses, dysuria, localized erythema, etc), patients who had received intravenous antibiotics before ED presentation, patients transferred from other hospitals (due to unavailable health records it was not possible to determine whether they received antibiotics), patients who presented with non-COVID-19 complaints (eg, trauma), and patients who had finished empirical oral antibiotic treatment for COVID-19 more than seven days ago (treatment was too long ago to affect the current presentation) or had started oral antibiotics in under 24 hours (not to preclude any potential antibiotic effects). Patients with several ED visits were excluded as reasons for several visits were unrelated to the study (eg, patients without primary health care providers in Zagreb, patients requiring specific procedures or testing that was only available at our institution for SARS-CoV-2 positive patients etc).

### Outcomes

The primary outcome was hospital admission. The secondary outcomes were pneumonia development, respiratory failure, and level of respiratory support required. Pneumonia was confirmed by x-ray or computed tomography scans. Respiratory failure was defined as SpO_2_<93%, pO_2_<8.00 kPa, or pCO_2_>6 kPa. Level of respiratory support was categorized as nasal cannula/mask oxygenation, high-flow nasal cannula (HFNC), or mechanical ventilation. Patients were oxygenated by mask or nasal cannula when SpO_2_ was below 92% for those without chronic obstructive pulmonary disease (COPD) and below 89% for those with COPD. HFNC was initiated when SpO_2_ was below 90% despite maximum oxygenation by mask (15L/min). Patients were intubated and mechanically ventilated when hypoxemia <90% persisted despite maximum HFNC settings in the ED, or when patient presentation required immediate intubation as per clinical judgment of the attending emergency medicine specialist.

### Statistical analysis

Categorical variables are presented as counts and percentages, and were compared between groups by using the χ^2^ test or Fisher exact test for counts <5. Continuous variables are presented as means and standard deviations (SD) or as medians where appropriate. For continuous and ordinal variables, the *t* test or Mann-Whitney test was used, where appropriate. All reported *P* values are two-sided and are considered to be statistically significant when *P* < 0.05. A propensity-score matching (PSM) method was used to reduce confounding (14). The selected covariates used in PSM were extracted based on literature review and selected based on available data (15). Logit PSM was performed by using the 1:1 nearest neighbor algorithm with a caliper distance of 0.1 without replacement; exact matching was used for the sex variable. No trimming was performed. Patient groups were matched according to age and comorbidities; exact matching was used for the sex variable. Logistic regression model with odds ratios for each matching variable is presented in Supplementary material 1(Supplementary material 1). There were no missing data for variables used for propensity score calculation. In a subanalysis, logistic regression was used to determine whether antibiotic treatment significantly predicted the same outcomes when adjusted for antithrombotic pre-ED treatment. Data manipulation and statistical analysis were performed by using the IBM SPSS, version 26.0 (https://www.ibm.com/analytics/spss-statistics-software) and Propensity Score Matching for SPSS, Version 3.0.4 (underlying packages MatchIt, optmatch, RItools, SparseM and cem) from R software, version 3.5.0 (*cran.r-project.org*) (16-25).

## RESULTS

**Figure 1:**
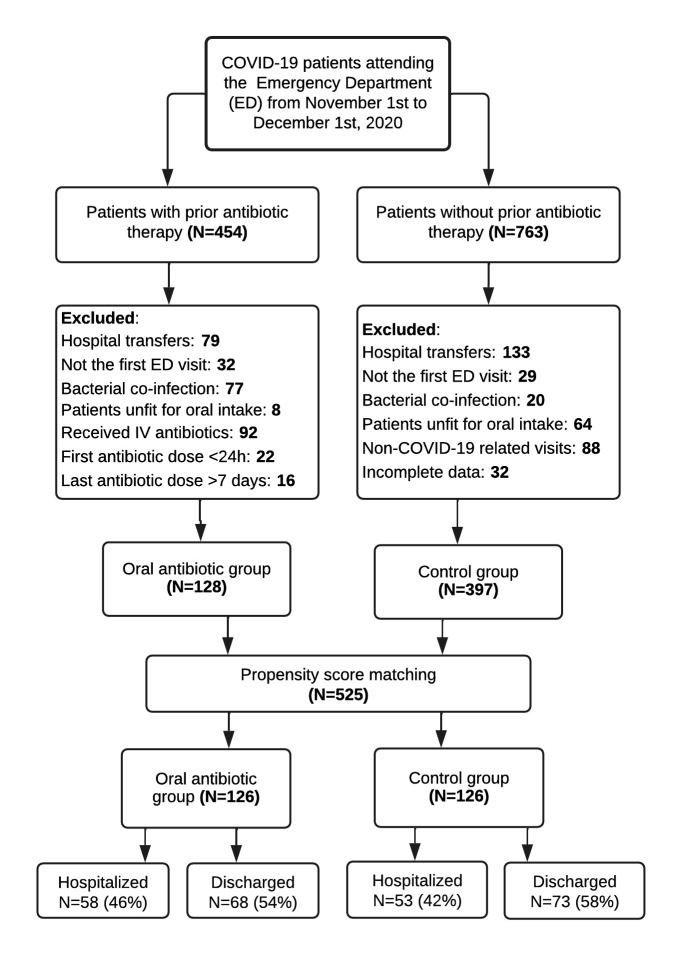
Flowchart of the study design. COVID-19 – coronavirus disease 2019.

After evaluating 1217 patient ED visits, 525 patients met the inclusion criteria, with 128 patients in the antibiotic treatment group and 397 in the control group. After calculating the propensity scores, 126 pairs were matched and 273 patients were excluded (Figure 1).

### Unmatched patient characteristics

The mean age was 63.5 years (SD 16.2); 60.8% patients were male and 355 (67.6%) had one or more comorbidities (Table 1). Mean symptom onset was 8.4 days (SD 4.2) before presentation to the ED. Most patients, 84%, received azithromycin, 30% received amoxicillin and clavulanic acid, and 9% received other antibiotics. More than two different antibiotics were prescribed to 18% of patients. Electronic health records of the prescribing physician were available in 65% of patients. In 85.5% of cases the primary care physician was reported as the prescribing physician. In the unmatched group, oral antibiotics were prescribed to younger patients with fewer comorbidities, particularly arterial hypertension, chronic kidney disease (CKD), atrial fibrillation, and cardiovascular disease (Table 1). Patients taking oral antibiotics presented to the ED one day later than controls. Physical examination findings, inflammatory markers, and radiographic findings did not significantly differ between the groups (Supplementary material 2(Supplementary material 2)). The observed difference in renal function indicators in the unmatched cohort can most likely be explained by more patients in the control group receiving renal replacement therapy. The significant difference persisted even after matching, but after excluding CKD patients from the analysis no significant difference was observed. Nine patients from both groups had a left shift in the complete blood counts. Overall, 272 patients (51.8%) were admitted to hospital (Table 1), 90% of whom required oxygen administration by mask or nasal cannula, 6% required HFNO, and 3% were intubated and mechanically ventilated on presentation (Table 2). Pneumonia developed in 431 patients (81.9%); 214 presented with respiratory failure (40.8%). Empirical oral treatment of COVID-19 before ED presentation did not affect pneumonia development, respiratory failure, hospital admission, required level of respiratory support, or average hospital stay (Table 2).

**Table 1:** Characteristics of controls and patients who received antibiotic treatment before admission to emergency department due to coronavirus disease 2019^‡^

	Patients							
	unmatched					matched		
Variable	treatment (n = 128)			control (n = 397)	d^†^	treatment (n = 126)	control (n = 126)	d^†^
Age*, years	59.6 (15.4)			64.7 (16.3)	-0.32	59.9 (15.3)	60.4 (15.3)	-0.03
Sex* (%)								0
female		50 (39.1)		156 (39.3)		48 (38.1)	48 (38.1)	
male		78 (60.9)		241 (60.7)		78 (61.9)	78 (61.9)	
Day of illness on presentation		9.4 (3.6)		8.1 (4.3)	0.34	9.4 (3.7)	8.8 (4.2)	0.15
Number of comorbidities, n (%)					-0.40			-0.02
0		59 (46.1)		111 (28.0)		59 (46.8)	55 (43.7)	
1		29 (22.7)		105 (26.4)		27 (21.4)	35 (27.8)	
2		26 (20.3)		92 (23.2)		26 (20.6)	20 (15.9)	
≥3		14 (10.9)		89 (22.4)		14 (11.1)	16 (12.7)	
**Comorbidities**								
Arterial hypertension, n (%)*		54 (42.2)	236 (59.4)		-0.35	53 (42.1)	54 (42.9)	0.02
Diabetes mellitus, n (%)*		24 (18.7)	102 (25.7)		-0.18	26 (20.6)	24 (19.0)	-0.04
Chronic kidney disease, n (%)*		3 (2.3)	29 (7.3)		-0.33	3 (2.4)	3 (2.4)	0.00
Chronic obstructive pulmonary disease/asthma, n (%)*		12 (9.4)	32 (8.1)		0.05	9 (7.9)	10 (7.9)	0.03
Hypothyroidism, n (%)*		9 (7.0)	32 (8.1)		-0.04	10 (7.9)	9 (7.1)	-0.031
Cardiovascular disease, n (%)*		4 (3.1)	50 (12.8)		-0.54	4 (3.2)	4 (3.2)	0.00
Cerebrovascular disease, n (%)*		3 (2.3)	23 (5.8)		-0.23	4 (4.8)	3 (2.4)	-0.05
Active cancer, n (%)		2 (1.6)	12 (3.0)		-0.12	2 (1.6)	2 (1.6)	0.00
Cancer in remission, n (%)*		7 (5.4)	26 (6.5)		-0.13	6 (4.8)	7 (5.6)	0.04
Atrial fibrillation (%)*		4 (3.1)	40 (10.1)		-0.40	5(4)	4 (3.2)	-0.04
**Medications**								
Angiotensin-converting enzyme inhibitor, n (%)		36 (28.1)	138 (35.0)		-0.14	36 (28.6)	37 (29.3)	-0.13
Warfarin, n (%)		2 (1.6)	32 (8.1)		-0.31	2 (1.6)	6 (5)	-0.18
Direct oral anticoagulants, n (%)		1 (0.8)	15 (3.1)		-0.20	1 (0.8)	2 (1.6)	-0.07
Statins, n (%)		16 (12.5)	85 (21.4)		-0.24	16 (12.7)	18 (14.2)	-0.05
Anti-platelet/anti-aggregation therapy, n (%)		13 (10.2)	66 (26.6)		-0.19	13 (10.3)	17 (13.5)	-0.10

*variables used as covariates in propensity score matching.

†standardized mean difference.

‡values are means and standard deviations unless indicated otherwise.

**Table 2:** Outcomes of controls and patients who received antibiotic treatment before admission to emergency department due to coronavirus disease 2019

	Patients										
	unmatched					matched					
Variable	treatment (n = 128)	control (n = 397)		P		treatment (n = 126)		control (n = 126)		P	
Primary outcome											
Hospital admission, n (%)	60 (46.9)	212 (53.4)		0.2		58 (46)		53 (42.1)		0.53	
Secondary outcomes											
Pneumonia (all imaging modalities), n (%)	112 (87.5)		319 (80.4)		0.07		108 (85.7)		96 (76.2)		0.054	
Respiratory failure, n (%)	47 (36.7)		167 (42.1)		0.28		47 (37.3)		45 (33.3)		0.89	
Respiratory support, n (%)											0.34	
None	68 (53.1)		185 (46.6)				68 (54)		73 (57.9)			
Supplementation by mask or nasal cannula	52 (40.6)		194 (48.9)				50 (39.7)		48 (38.1)			
High flow nasal oxygenation	6 (4.7)		11 (2.7)				6 (4.8)		3 (2.4)			
Mechanical ventilation	2 (1.5)		7 (1.8)				2 (1.6)		2 (1.6)			

### Propensity score matching

**Figure 2:**
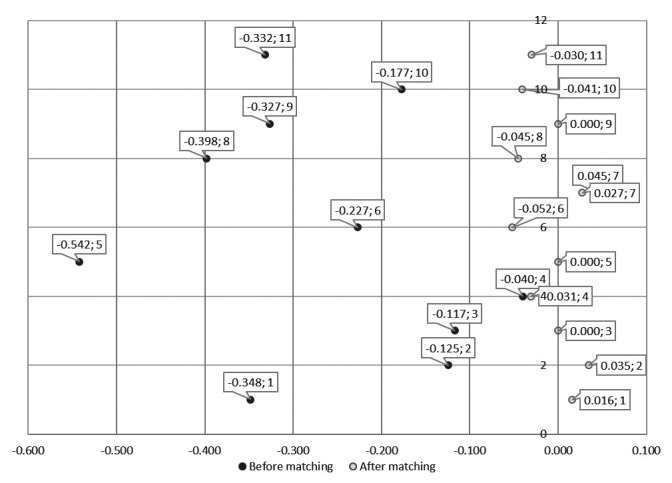
Dot plot of standardized mean differences before and after propensity score matching for matching variables showing robust matching. 1 – arterial hypertension, 2 – cancer in remission, 3 – active cancer, 4 – hypothyroidism, 5 – coronary heart disease, 6 – cerebrovascular disease, 7 – chronic obstructive pulmonary disease/asthma, 8 – atrial fibrillation, 9 – chronic kidney disease, 10 – diabetes mellitus, 11 – age.

The 126 matched pairs demonstrated a good balance between the groups. Overall covariate balance after matching was tested by Hansen & Bowers test (χ^2^ = 5.494, *P* = 0.856). Standard differences in the mean propensity scores between two groups before and after matching displayed no significant imbalance, indicating robust matching (Table 1, Figure 2) (14). The largest standardized mean difference in absolute value for any given variable was 0.052, which is lower than the upper limit of 0.25 recommended by Rubin and Stuart (26,27). Before matching, the treatment and control group significantly differed in age, hypertension, CKD, cardiovascular disease, and atrial fibrillation. Hence, significant differences before matching were observed in the rates of statin and warfarin use. After matching, no significant differences in the variables selected for propensity score matching were observed (Figure 2, Supplementary material 3(Supplementary material 3), Supplementary material 4(Supplementary material 4), Supplementary material 5(Supplementary material 5)).

### Matched patients' characteristics

In the matched group, the mean age was 60.2 years (SD 15.3); 61.9% of patients were men and 138 (55%) had one or more comorbidities. The mean number of days since symptom onset was 9.1 days (SD 3.8), with no significant difference between the groups (Table 1). For both groups, leukocyte count was within the reference range. C-reactive protein levels were elevated, without significant difference between the groups. On average, other baseline laboratory values were within the reference ranges and without significant difference between the groups (Supplementary material 2(Supplementary material 2)).

In the matched group, 111 (44%) patients were admitted, 92 (36.5%) presented with respiratory failure, and 204 (81%) presented with pneumonia. On admission, 98 (38.9%) patients required supplemental oxygen by mask, 9 (3.6%) required HFNO, and 4 (1.6%) were intubated on presentation. Prior oral antibiotic treatment did not significantly affect vital signs, respiratory failure, pneumonia development, requirement of respiratory support, or hospital admission (Table 2).

When adjusted for antithrombotic pre-ED treatment in a binominal logistic regression model, antibiotic treatment was associated with a higher prevalence of pneumonia (odds ratio 2.04, 95% confidence interval 1.025-4.062, *P* = 0.04, Nagelkerke R^2^ = 0.08, χ^2^ = 2.03). Other outcomes, when adjusted for antithrombotic pre-ED treatment, remained non-significant (Supplementary material 6(Supplementary material 6)).

## DISCUSSION

Our retrospective study with PSM of 525 ED patients displayed no benefit of empirical oral antibiotic treatment for COVID-19. In the unmatched cohorts, the treated group had somewhat fewer hospital admissions than controls, but in the matched groups there was no relevant difference. Considering the secondary outcomes in unmatched sets, the treated group showed a tendency for pneumonia development and lesser requirement of respiratory support. After matching, however, no consistent benefit of outpatient oral antibiotics in the treatment of COVID-19 was observed. Yet, an almost one third (28%) of all patients who presented to the ED for COVID-19 in the studied period reported using antibiotics. The observed antibiotic prescription rates agree with those from two US EDs (28). Increased antibiotic use is observed without evidence of a treatment benefit, which in the long term could lead to increased antibiotic resistance (29).

This article presents real-life data from a cross-section of typical COVID-19 adult patients of all ages. We believe that our exclusion criteria favored younger patients, as patients previously hospitalized or unfit for oral intake were more likely to be older. In other studies, the most commonly evaluated antibiotic is azithromycin (12,13,30,31), which was the most frequently reported antibiotic in our study. A large RCT from the UK, the PRINCIPLE trial, assessed azithromycin as treatment for suspected COVID-19 in the outpatient setting (12). Comparable to our results, they reported no justification for azithromycin use in the treatment of COVID-19. They reported no effect of azithromycin on hospital visit rates, hospital admissions, or mortality vs usual care for suspected COVID-19. Oral azithromycin did not reduce the time to full recovery. Importantly, the PRINCIPLE trial included only adults older than 65 or those older than 50 with comorbidity, while our study included all adult patients. Another RCT found no effect of outpatient single-dose oral azithromycin on disease severity, disease duration, or hospital admission rates (13). The study showed increased ED visit rates by patients who were taking azithromycin vs placebo, possibly due to the increased occurrence of symptoms attributed to azithromycin side effects, such as diarrhea, nausea, and vomiting.

Drawbacks of this study, particularly for evaluating ED-specific outcomes, were the low number of patients enrolled, with only 19 patients seeking ED attention, inclusion of asymptomatic individuals, and exclusion of all individuals older than 55. When compared with studies at the outpatient level, our study enabled a more precise evaluation of specific outcomes. Studies on hospitalized patients added further evidence against the use of antibiotics for treating COVID-19. The RECOVERY trial, involving 7763 admitted patients, reported no benefit of in-hospital azithromycin on hospital stay, mechanical ventilation requirement, or mortality. Several RCTs evaluating in-hospital antibiotic treatment in hospitalized patients consistently reported no mortality benefit (30-32). A retrospective study demonstrated that early administration of antibiotics to critically ill patients had no mortality benefit (1).

Our study included only confirmed COVID-19 patients who started their antibiotic treatment at the primary-care level. Confounding was handled by strict exclusion criteria and PSM. Another limitation of this study is the single-center setting. However, because the hospital was designated as a COVID-19 hospital serving around one million inhabitants, we believe that our sample comes from a diverse population. Presumably, patients visiting the ED have more severe symptoms than patients treated in the outpatient setting and represent a different population sample. A further limitation is the lack of data regarding risk factors, including weight and smoking status. Due to the low number of patients requiring HFNC or mechanical ventilation, our study is underpowered to draw conclusions on these outcomes.

In conclusion, our findings demonstrate no benefit of empirical antibiotic use in the outpatient treatment of COVID-19 in adults, including no difference in hospital admissions, pneumonia development, respiratory failure, or level of respiratory support required. Our findings suggest avoiding inappropriate prescribing of antibiotics in the treatment of COVID-19 in the community.
