# Supplementary material for: The effect of outpatient antibiotic treatment of coronavirus disease 2019 on the outcomes in the emergency department: a propensity score matching study
Source: Croat Med J. 2022 Feb;63(1):53–61. doi: 10.3325/cmj.2022.63.53 (PMC8895338; doi:10.3325/cmj.2022.63.53)
Supplement: Supplementary Material 1 [file CroatMedJ_63_s001.pdf]

Supplementary Table 1 - Logistic regression model with variables used for propensity score matching

Variable	B coefficient	Standard error	p	Odds Ratio	95% Confidence interval	
					Lower	Upper
Age	-0.011	0.008	0.122	0.989	0.979	1.009
Female	0.047	0.224	0.835	1.048	0.677	1.632
Diabetes mellitus	0.074	0.276	0.788	1.077	0.525	1.661
Chronic kidney disease	0.886	0.639	0.166	2.425	0.646	8.017
Atrial fibrillation	0.744	0.560	0.184	2.105	0.657	5.950
Chronic obstructive pulmonary disease	-0.358	0.372	0.335	0.699	0.342	1.477
Cerebrovascular disease	0.504	0.648	0.437	1.655	0.424	5.445
Cardiovascular disease	1.177	0.546	0.031	3.244	1.078	9.190
Hypothyroidism	0.182	0.411	0.659	1.199	0.520	2.623
Active cancer	0.439	0.843	0.603	1.550	0.280	7.691
Cancer in remission	0.243	0.477	0.611	1.275	0.540	3.550
Hypertension	0.388	0.262	0.139	1.473	0.882	2.462
Constant	-4.321	1.628	0.008	0.016		

Model summary Nagelkerke R2 = 0.078, chi-square= 8.25, p= 0.41.
