# Supplementary material for: The effect of outpatient antibiotic treatment of coronavirus disease 2019 on the outcomes in the emergency department: a propensity score matching study
Source: Croat Med J. 2022 Feb;63(1):53–61. doi: 10.3325/cmj.2022.63.53 (PMC8895338; doi:10.3325/cmj.2022.63.53)
Supplement: Supplementary Material 2 [file CroatMedJ_63_s002.pdf]

Supplementary Table 2 – Physical examination and laboratory findings

	UNMATCHED			MATCHED		
**Variable**	**Treatment (N=128)**	**Control (N=397)**	**d value**	**Treatment (N=126)**	**Control (N=126)**	**d**
	**PHYSICAL EXAMINATION**					
Altered mental state, N (%)	2 (1.6)	14 (3.5)	-0.12	2 (1.6)	4 (3.2)	-0.10
Fever (T >37.5°C), N (%)	15 (21.7)	55 (23.9)	-0.06	15 (11.9)	15 (11.9)	0
Respiratory failure, N (%)	47 (36.7)	167 (42.1)	-0.11	47 (37.3)	45 (35.7)	0.03
Pulse, beats per min	94 (15)	89 (18.4)	0.28	93.7 (15.1)	89.3 (17.4)	0.27
Dyspnoea			0.05			0.12
No dyspnoea (%)	90 (70.3)	289 (72.8)		88 (69.8)	97 (77)	
Respiratory rate 20-30/min (%)	29 (22.7)	82 (20.7)		29 (23)	23 (18.3)	
Respiratory rate >30/min (%)	9 (7.0)	26 (6.5)		9 (7.2)	6 (4.8)	
Systolic BP, mmHg	131 (19.5)	132 (23.1)	-0.05	131 (19.6)	135.1 (20.6)	-0.20
Diastolic BP, mmHg	82 (12.7)	80 (12.9)	0.14	81.9 (12.7)	83.4 (12.8)	-0.12
	**LABORATORY FINDINGS**					
Leukocyte count, $\times 10^9$/L	8.04 (3.8)	7.2 (3.5)	0.09	8.1 (3.8)	7.0 (3.1)	0.31
Erythrocyte count, $\times 10^9$/L	4.7 (0.73)	4.7 (0.59)	0.19	4.7 (0.7)	4.6 (0.5)	0.12
Haemoglobin, g/L	137.8 (18)	136.4 (17)	0.11	138 (18.6)	138.7 (15.9)	-0.04
MCV, fL	86.9 (4.3)	88.2 (5.7)	0.08	86.9 (4.4)	88.2 (7.2)	-0.22
RDW, %	13.5 (1.4)	13.8 (1.3)	-0.02	13.5 (1.4)	13.5 (1.2)	0.00
Thrombocytes, $\times 10^{12}$/L	242 (103.0)	221 (98.5)	0.24	242 (103)	220 (97)	0.22
MPV, fL	8.9 (1.11)	9.1 (1.01)	0.01	8.9 (1.1)	8.9 (1.1)	0.07
Neutrophils (%), %	75.8 (11.6)	74.1 (12.5)	0.14	75.8 (11.7)	72.1 (11.6)	0.06
Lymphocytes (%), %	15.8 (9.1)	16.8 (9.7)	-0.09	15.8 (9.2)	18.4 (9.1)	0.12
Neutrophil count, $\times 10^9$/L	6.3 (3.8)	5.78 (3.16)	0.15	6.3 (3.8)	5.2 (2.9)	0.17
Lymphocyte count, $\times 10^9$/L	3.81 (0.56)	6.34 (1.4)	-0.08	1.1 (0.6)	1.1 (0.5)	0.17
eGFR, mL/min $\times 1.76m^2$	85.2 (22.7)	73.7 (27.9)	0.35	84.7 (22.6)	81.9 (23.6)	0.08
Creatinine, µmol/L	81.0 (24.5)	103.2 (74.3)	-0.38	81.2 (24.6)	85.4 (32.7)	0.04
Urea, mmol/L	6.7 (4.0)	8.7 (6.3)	-0.38	6.6 (4.0)	6.6 (5.1)	0.13
Glucose, mmol/L	7.7 (3.4)	8 (3.5)	-0.06	7.8 (3.5)	7.8 (3.3)	0.10
AST, U/L	48 (32.5)	51 (44)	-0.07	48.6 (32.5)	45.7 (32.7)	0.18
ALT, U/L	46 (37.7)	46 (61.1)	0.01	46.9 (37.8)	43.2 (53.3)	0.13
LDH, U/L	359 (168)	344 (159)	0.33	361.2 (168.6)	320.2 (128.3)	0.32
CK, U/L	176 (201)	197 (244)	0.11	180.4 (203.5)	193 (195.3)	0.21
GGT, U/L	77 (116.3)	64 (70.4)	0.11	78.5 (117.2)	65.1 (71)	0.12
ALP, U/L	89 (100)	80 (50)	0.10	89.6 (101.6)	75.4 (49.6)	0.03
Na, mmol/L	136 (4.25)	136 (4.37)	-0.07	136 (4.3)	136.2 (3.8)	0.22
K, mmol/L	4.3 (0.45)	4.2 (0.51)	-0.03	4.3 (0.5)	4.2 (0.5)	0.24
CRP, mg/L	87.2 (77.5)	89.9 (80.6)	-0.02	88.2 (77.6)	81.3 (78.8)	0.09
SpO_2, %	92.1 (5.9)	91.3 (8.9)	0.14	92.0 (6.0)	91.5 (10.6)	-0.13
PaO_2, kPa	9.02 (2.7)	8.99 (2.9)	0.13	9.0 (2.8)	9.2 (2.7)	-0.15
$PaCO_2$, kPa	4.58 (0.8)	4.6 (1.4)	0.09	4.6 (0.8)	4.5 (0.8)	-0.11
pH (arterial)	7.459 (0.05)	7.455 (0.07)	0.14	7.459 (0.054)	7.465 (0.052)	-0.14
HCO_3, mmol/L	25.1 (3.1)	24.7 (3.5)	0.15	25.1 (3.1)	25 (2.7)	-0.13

All values are reported as means (SD), unless indicated otherwise.
d value - standardized mean difference
