# Supplementary figures and images for: The effect of outpatient antibiotic treatment of coronavirus disease 2019 on the outcomes in the emergency department: a propensity score matching study
Source: Croat Med J. 2022 Feb;63(1):53–61. doi: 10.3325/cmj.2022.63.53 (PMC8895338; doi:10.3325/cmj.2022.63.53)

**Unmatched Treated**

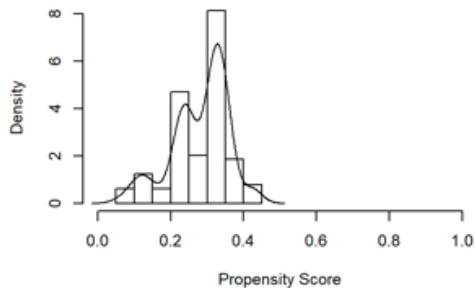

**Matched Treated**

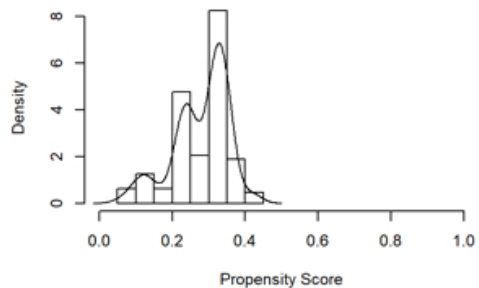

**Unmatched Control**

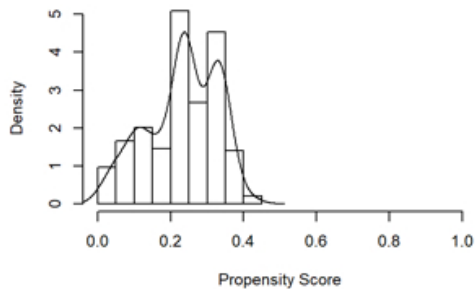

**Matched Control**

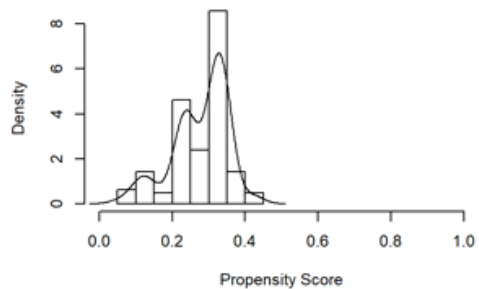

Supplement: Supplementary Material 3 [file CroatMedJ_63_s003.pdf]

## Standardized differences before matching

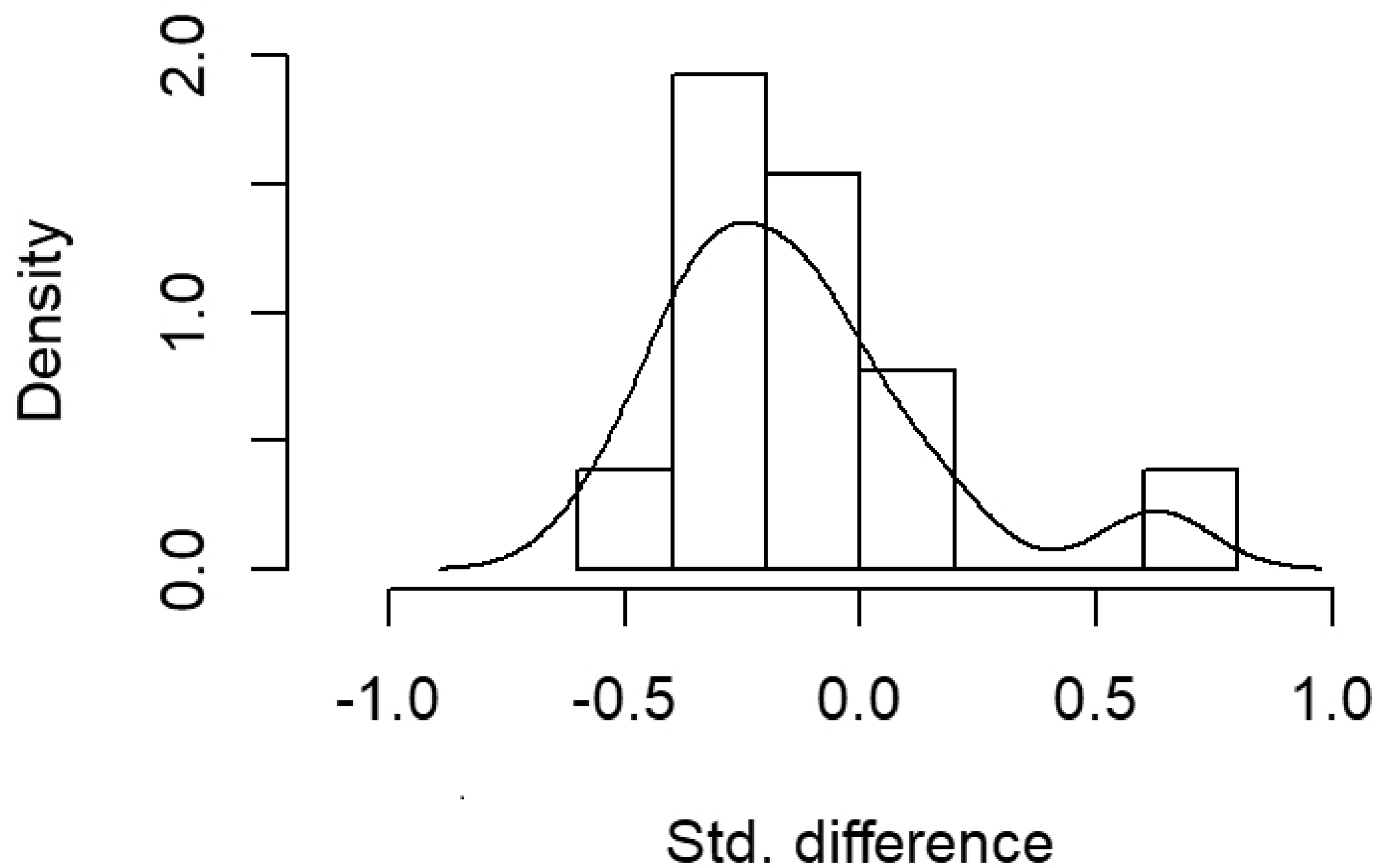

## Standardized differences after matching

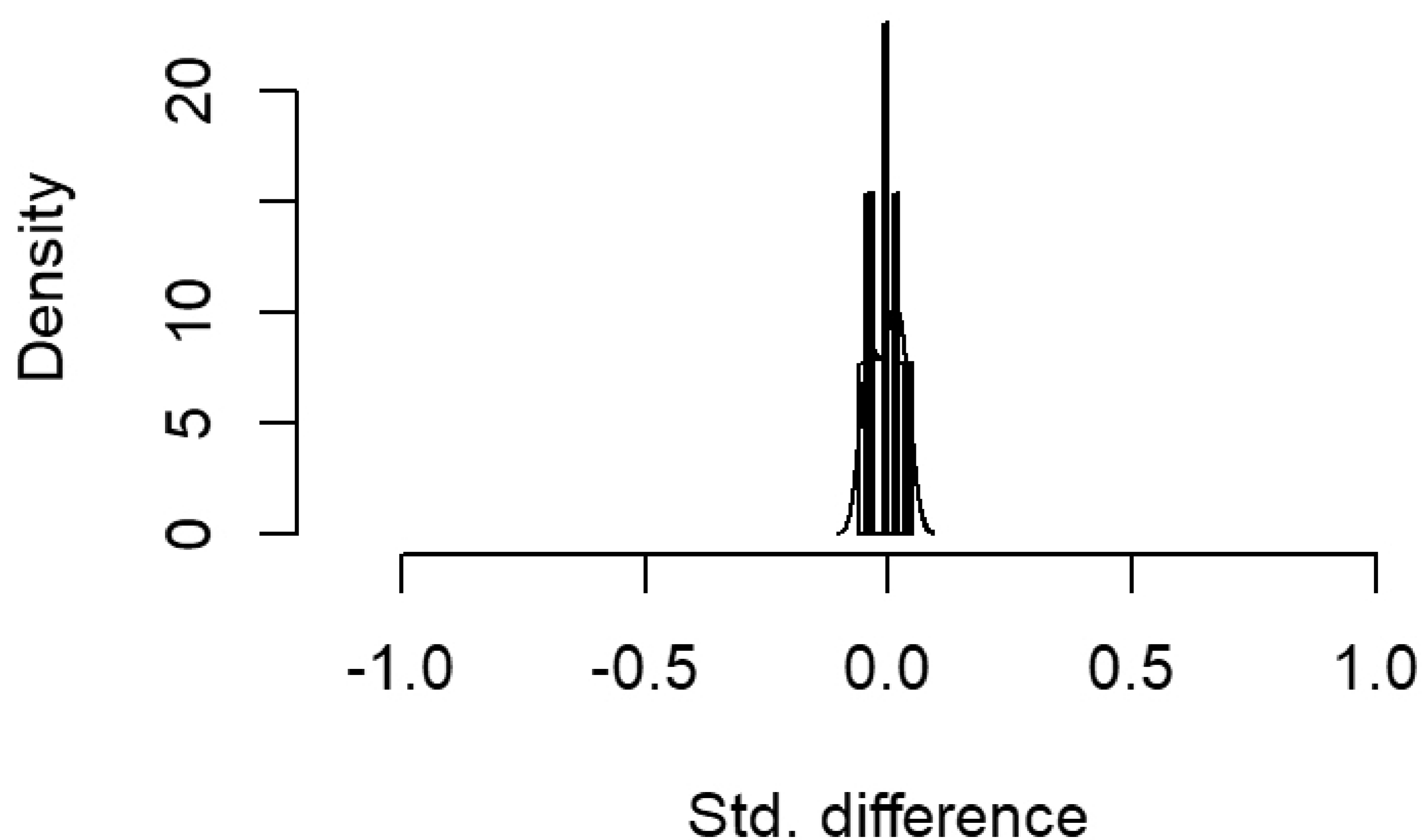

Supplement: Supplementary Material 4 [file CroatMedJ_63_s004.pdf]

# Distribution of Propensity Scores

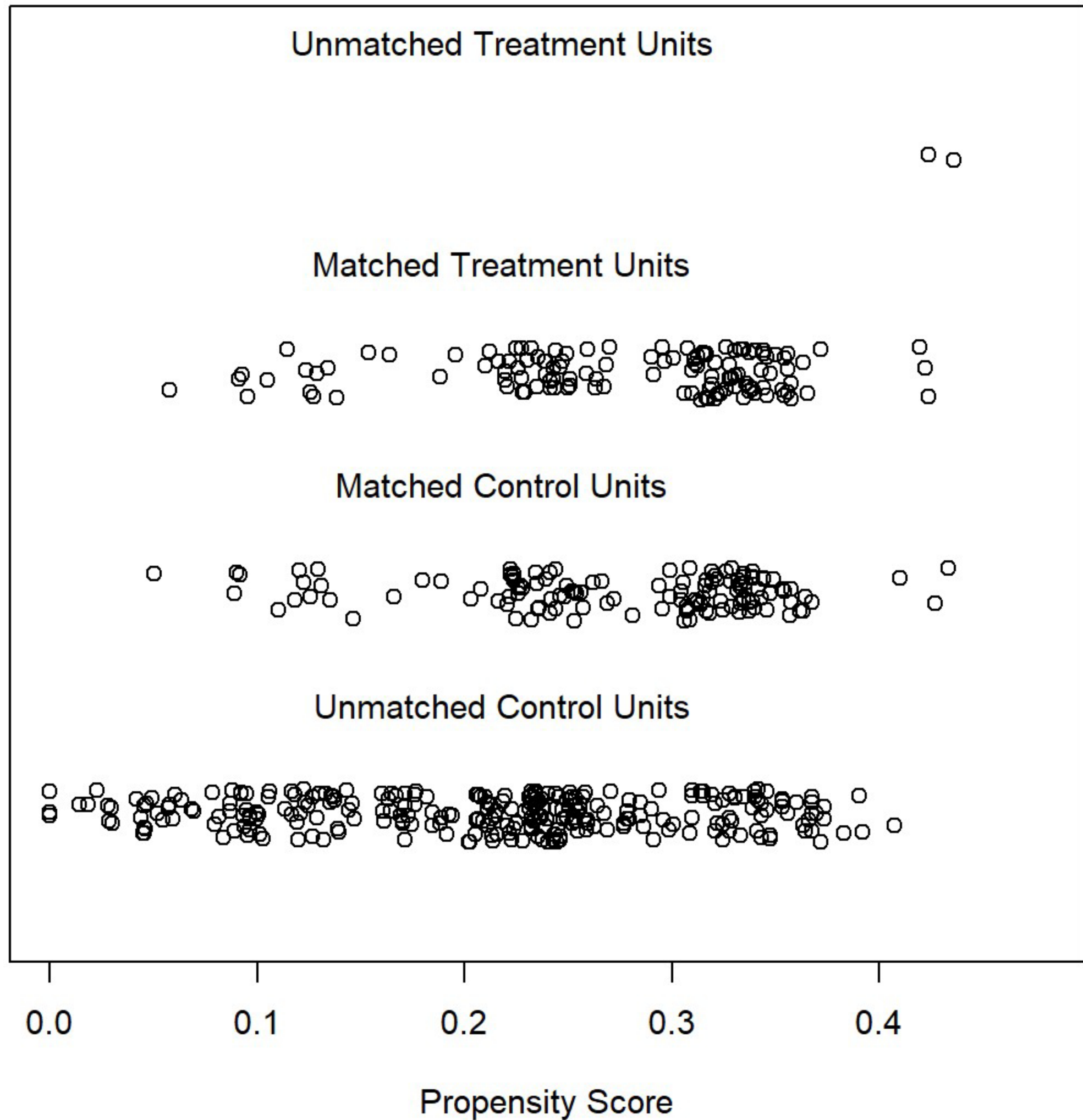

Supplement: Supplementary Material 5 [file CroatMedJ_63_s005.pdf]
