# Supplementary material for: The effect of outpatient antibiotic treatment of coronavirus disease 2019 on the outcomes in the emergency department: a propensity score matching study
Source: Croat Med J. 2022 Feb;63(1):53–61. doi: 10.3325/cmj.2022.63.53 (PMC8895338; doi:10.3325/cmj.2022.63.53)
Supplement: Supplementary Material 6 [file CroatMedJ_63_s006.pdf]

Supplementary Table 3 – Differences in outcomes between treated and controls in the matched cohort adjusted for prior antithrombotic/antiplatelet medication

Outcome	B	Standard error	p	Odds ratio	95% Confidence Interval	
					Lower	Upper
Hospital admission	0.200	0.258	0.438	1.222	0.737	2.027
Pneumonia	-0.713	0.351	0.042	2.042	1.025	4.062
Respiratory failure	0.097	0.264	0.715	1.101	0.656	1.849
Respiratory support, none	reference		0.674			
HFNO	-0.007	1.025	0.995	0.993	0.133	7.400
Supplementation by mask or nasal cannula	0.726	0.743	0.328	2.066	0.482	8.860
Mechanical ventilation	-0.149	0.266	0.577	0.862	0.511	1.452

HFNO – High Flow Nasal Oxygenation
